# Circulating Exosomal miR-96 as a Novel Biomarker for Radioresistant Non-Small-Cell Lung Cancer

**DOI:** 10.1155/2021/5893981

**Published:** 2021-02-27

**Authors:** Qin Zheng, Huaiyin Ding, Lixue Wang, Yu Yan, Yuan Wan, Yongxiang Yi, Liang Tao, Chuandong Zhu

**Affiliations:** ^1^Department of Oncology, Nanjing Second Hospital, Nanjing University of Chinese Medicine, Nanjing 210003, Jiangsu, China; ^2^Department of Oncological Radiotherapy, Nanjing Second Hospital, Nanjing University of Chinese Medicine, Nanjing 210003, Jiangsu, China; ^3^Department of Radiology, Nanjing Second Hospital, Nanjing University of Chinese Medicine, Nanjing 210003, Jiangsu, China; ^4^The Pq Laboratory of Micro/Nano BiomeDx, Department of Biomedical Engineering, Binghamton University—SUNY, Binghamton, NY 13902, USA; ^5^Department of General Surgery, Nanjing Second Hospital, Nanjing University of Chinese Medicine, Nanjing 210003, Jiangsu, China; ^6^Department of Gastrointestinal Surgery, Nanjing Drum Tower Hospital, The Affiliated Hospital of Nanjing University Medical School, Nanjing 210008, Jiangsu, China

## Abstract

Patients with non-small-cell lung cancer (NSCLC) frequently develop radioresistance, resulting in poor response to radiation and unfavourable prognosis. Early detection of radioresistance hence can guide the adjustment of treatment regimens in time. Exosomes are lipid bilayer-enclosed vesicles with sub-micrometer size that are released by various cells. Exosomes contain a tissue-specific signature wherein a variety of proteins and nucleic acids are selectively packaged. Growing evidence shows exosomes are involved in cancer pathophysiology and exosomes as the latest addition to the liquid biopsy portfolio have been used in cancer diagnosis. Compared to cell free RNA, exosomal lipid envelope can effectively protect RNA cargo against degradation. Therefore, exosomes may hold great promise for the identification of radioresistance. Here, we report six plasma exosomal miRNAs could be used to distinguish radioresistant NSCLC patients from radiosensitive NSCLC patients and to evaluate the prognosis of NSCLC. Samples were obtained from 52 NSCLC patients with or without radioresistance and 45 age-matched healthy volunteers. Exosomes in 1 ml plasma were isolated followed by extraction of small RNA. The expression levels of miRNAs were determined by quantitative real-time PCR. Potential miRNA markers were further evaluated in additional 52 NSCLC patients. We found exosomal miR-1246 and miR-96 are significantly overexpressed in NSCLC patients. Moreover, exosomal miR-96 in patients with radioresistant NSCLC is significantly higher than that of controls. Exosomal miR-96 also demonstrates a significant correlation with vascular invasion and poor overall survival. Altogether, our results indicate that exosomal miR-96 could be a non-invasive diagnostic and prognostic marker of radioresistant NSCLC.

## 1. Introduction

Lung cancer is the leading cause of cancer-related death worldwide [1]. More than 2.3 million people are newly diagnosed with lung cancer every year, and non-small-cell lung cancer (NSCLC) accounts for approximately 80% of all cases of lung cancer [2]. Most of the NSCLC patients receive radiotherapy throughout the course of their diseases [3, 4]. However, clinical studies have revealed that only some NSCLC patients benefit from radiotherapy, while more NSCLC patients are radioresistant and suffer radiotherapy failure [5]. Furthermore, these radioresistant NSCLC patients are prone to fatal complications such as radiation pneumonitis and radiation oesophagitis [6, 7]. Biomarkers that can distinguish radioresistant patients from radiosensitive patients among NSCLC can spare some patients from the pain of radiotherapy complications and reduce the waste of medical resources and time, while improving the control of lung cancer radiotherapy.

MicroRNAs (miRNAs), which play significant roles in the diagnosis and prognosis of many diseases [8, 9], are considered candidates for blood-based biomarkers of radioresistant NSCLC patients. Several studies have demonstrated that circulating miRNAs such as miR-21, miR-1246, let-7g, miR-210, miR-214, and miR-96 have a close relationship with radioresistance of NSCLC cell lines or xenograft models [10–13]. Moreover, circulating miRNAs have been extensively investigated for their important roles in non-invasive diagnosis.

Exosomes are 30–140 nm nanovesicles secreted by cells that contain proteins and nucleic acids, such as miRNAs and DNAs [14]. Unlike circulating miRNAs, miRNAs encapsulated in exosomes are resistant to RNases and are stable in the blood; hence, exosomal miRNAs are considered to be more promising candidates than circulating miRNAs as novel biomarkers of many cancers [15, 16]. Plasma exosomal miRNAs are being developed as diagnostic, prognostic, and even therapeutic biomarkers in patients with various tumour types, such as prostate cancer [17], multiple myeloma [18], and lung cancer [11]. Nonetheless, the diagnostic value of exosomal miRNAs for radioresistant NSCLC remains unknown, and new exosomal miRNA markers need to be verified.

In this follow-up study, we selected six circulating miRNAs (miR-21, miR-1246, let-7g, miR-210, miR-214, and miR-96) that have been reported to be dysregulated and closely correlated with radioresistance in NSCLC. The aim of this study was to investigate the feasibility of using six plasma exosomal miRNAs as novel biomarkers to distinguish radioresistant NSCLC patients from radiosensitive NSCLC patients and to further examine their correlation with the prognosis of NSCLC. We used the quantitative real-time polymerase chain reaction (qRT-PCR) method to measure the exosomal miRNAs in 52 NSCLC patients (27 radioresistant NSCLC patients and 25 radiosensitive NSCLC patients) and 45 healthy volunteers. The results demonstrated that exosomal miR-96 is a promising diagnostic biomarker for radioresistance in NSCLC and is associated with poor prognosis of NSCLC.

## 2. Methods

### 2.1. Patients

A total of 52 NSCLC patients who received radiotherapy and 45 healthy volunteers at Nanjing Second Hospital, Nanjing University of Chinese Medicine University, were enrolled in our study from June 2010 to July 2014. According to radiotherapy outcomes, the NSCLC patients were divided into two groups: a radioresistant patient group (RRP, *n* = 27) and a radiosensitive patient group (RSP, *n* = 25), and healthy volunteers (HVs, *n* = 45) were used as a control. Subjects in each group were matched for factors such as age, gender, and smoking. The clinical characteristics of the 52 NSCLC patients and 45 HVs are shown in Table 1. The studies were conducted in accordance with International Ethical Guidelines for Biomedical Research Involving Human Subjects (CIOMS), and the use of patient samples was approved by the Ethics Committee of Nanjing Second Hospital, Nanjing University of Chinese Medicine. All participants understood the study premise and signed an informed consent form. All patients were histologically diagnosed, and their tumour stages were determined according to the International Union against Cancer (UICC) guidelines.

### 2.2. Samples

Blood samples (1 mL) were collected in green-top vacutainer tubes. Samples were centrifuged at 1500 × g for 15 minutes at 4°C, and the supernatants were transferred to fresh tubes and stored at −80°C. Before being processed, the plasma samples were filtered through a 0.45 *μ*m pore membrane (Millipore, Billerica, MA, USA).

### 2.3. Isolation of Exosomes from Plasma Using a Lipid Nanoprobe

Plasma exosomes were isolated by a lipid nanoprobe (LNP) system as previously described [19]. Briefly, aliquots of 100 nmol of labelled probes (LPs) in 500 *μ*l of Diluent C (Sigma-Aldrich Co., USA, No. CGLDIL) were added to 100 *μ*l of plasma and then mixed for 5 min at 4°C and incubated with capture probes (CPs) at room temperature for 10 min. Additionally, 100 *μ*l of plasma was mixed with 30 ml PBS and ultracentrifuged once at 100,000 g and 4°C for 2 h. Captured exosomes were released using biotin. DSPE-PEG-desthiobiotin (Nanocs) in pure anhydrous ethanol was prepared as above. Following the above-mentioned protocol, nEVs were labelled with DSPE-PEG-desthiobiotin and captured onto CPs. Non-captured exosomes were removed by rinsing the CPs three times with PBS. Twenty nanomoles of biotin in PBS was introduced to displace the DSPE-PEG-desthiobiotin. After incubation for 30 min at room temperature, CPs were thoroughly washed with PBS using a pipette. The supernatant was collected for RNA extraction. Release efficiency was calculated by the amount of RNA extracted from the supernatant (released exosomes) divided by the total amount of RNA from captured exosomes.

### 2.4. Transmission Electron Microscope (TEM)

Five microliters of exosomes was spotted on a 400-mesh Formvar-coated copper grid on filter paper, which was subsequently dried for 20 min using an infrared lamp. After the liquid was removed by the filter paper, the grid was negatively stained with 1% uranyl acetate for 1 min and then air-dried at room temperature. Exosomes were examined under FEI Tecnai TEM (JEOL, Hitachi, Japan, No. JSM-6360LV) operated at 100 kV.

### 2.5. Western Blot Analysis

Isolated exosomes were lysed using RIPA buffer (Sigma-Aldrich Co., USA, No. R0278) containing 50 mM Tris-HCl, pH 8.0, 150 mM sodium chloride, 1.0% Igepal, 0.5% sodium deoxycholate, 0.1% sodium dodecyl sulfate, and a protease inhibitor cocktail. The total protein concentration was normalized by the Bradford assay. Wet electrophoretic transfer was used to transfer the proteins onto a polyvinylidene membrane. The membrane was blocked with 5% non-fat dry milk in PBS and incubated with 0.05% Tween 20 for 1 h at room temperature and then incubated with primary antibodies against CD63 (Santa Cruz Biotechnology, sc-15363) and Hsp70 (SBI, EXOAB-KIT-1) overnight at 4°C. Afterwards, fluorescence-labelled secondary antibodies were incubated for 1 h at room temperature. Samples were washed three times with PBS and 0.05% Tween 20 for 10 min. Blots were evaluated by the chemiluminescence method. An exosome standard (100 *µ*g, Human Plasma, BioVision, Inc. No. M1067) was used as a positive control.

### 2.6. NanoSight Analysis

Size distribution and quantification of exosomes were analysed using a NanoSight NS300 instrument (Malvern Instruments Ltd., Worcestershire, UK, No. MAN0516-07-EN-00). For NanoSight analysis, the exosome pellet was resuspended in 500 *μ*L sterile PBS, which was filtered with a 0.22 *μ*m syringe filter (Millipore). Samples were diluted (1 : 4000) until individual nanoparticles could be tracked. The size distribution and vesicle concentration were analysed with NTA software.

### 2.7. RNA Isolation from Exosomes

The miRNAs were extracted from plasma-isolated exosomes and plasma using a miRNeasy Micro Kit (QIAGEN, Valencia, CA) following the standard protocol from the manufacturer. The extracted RNA was eluted with 14 *μ*L of DNase-free, RNase-free water. The quantity and quality of RNA were determined by the Agilent Bioanalyzer 2100 using Small RNA Chip (Agilent Technologies, Santa Clara, CA) according to the manufacturer's instructions.

### 2.8. Quantitative Real-Time PCR

MiR-21, miR-1246, let-7g, miR-210, miR-214, and miR-96 (QIAGEN, Valencia, CA) were selected for validation. Cel-miR-39 **(**5'-UCACCGGGUGUAAAUCAGCUUG-3') served as an endogenous normalizer [20]. The primer sequences were as follows: miR-21 (sense): 5'-ACACTCCAGCTGGGTAGCTTATCAGACTGA-3' and miR-21 (antisense): 5'-CTCAACTGTGGTCGTGGAGTCGGCAATTCAGTTGAGTCAACCTC-3'; miR-1246 (sense): 5'-ACACTCCAGCTGGGAATGGATTTTTTGG-3' and miR-1246 (antisense): 5'-CTCAACTGGTGTCGTGGAGTCGGCAATTCAGTTGAGCCTGCTCC-3'; let-7g (sense): 5'-ACACTCCAGCTGGGTGAGGTAGTAGTTTGT-3' and let-7g (antisense): 5'-CTCAACTGGTGTCGTGGAGTCGGCAATTCAGTTGAGAACTGTAC-3'; miR-210 (sense): 5'-ACACTCCAGCTGGGAGCCCCTGCCCACCG-3' and miR-210 (antisense): 5'-CTCAACTGGTGTCGTGGAGTCGGCAATTCAGTTGAGCAGTGTGC-3'; miR-214 (sense): 5'-ACACTCCAGCTGGGTGCCTGTCTACACTTG-3' and miR-214 (antisense): 5'-CTCAACTGGTGTCGTGGAGTCGGCAATTCAGTTGAGGCACAGCA-3'; miR-96 (sense): 5'-ACACTCCAGCTGGGTTTGGCACTAGCACATT-3' and miR-96 (antisense): 5'-CTCAACTGGTGTCGTGGAGTCGGCAATTCAGTTGAGAGCAAAAA-3'. Five ng of the isolated exosomal RNA was reverse-transcribed using a QIAGEN miScript II RT kit with HighFlex buffer and universal RT primer. Five microliters of diluted cDNA products was used in a 10 *μ*L real-time PCRs containing 5 *μ*L QIAGEN SYBR green Master Mix, 1 *μ*L universal downstream primer, and 1 *μ*L miRNA-specific upstream primer. PCR was performed on an ABI ViiA 7 platform. The above mixture was incubated at 37°C for 60 min, 94°C for 5 min, and 4°C for 10 min. Relative quantification of miR expression was calculated using the 2^−ΔΔCt^ method.

### 2.9. Statistical Analysis

Data are presented as the mean ± SD. A Student *t*-test was used to compare differences in the miRNA expression level between NSCLC with HVs and RRP with RSP. A 2-sided *t*-test (unpaired or paired) and one-way ANOVA were used to compare differences in the clinical characteristics of the patients. Receiver-operating characteristic (ROC) curves and the area under the ROC curve (AUC) were used to calculate sensitivity and specificity and thereby to assess the accuracy of the diagnosis. In survival analysis, the probability of overall survival (OS) was determined by the Kaplan–Meier method with a log-rank test. All graphs were generated using GraphPad Prism 6, and statistical analysis was performed by SPSS 17.0 statistical software. A *p*-value <0.05 was considered statistically significant.

## 3. Results

### 3.1. Identification and Quantification of Exosomes in Plasma

**Figure 1:**
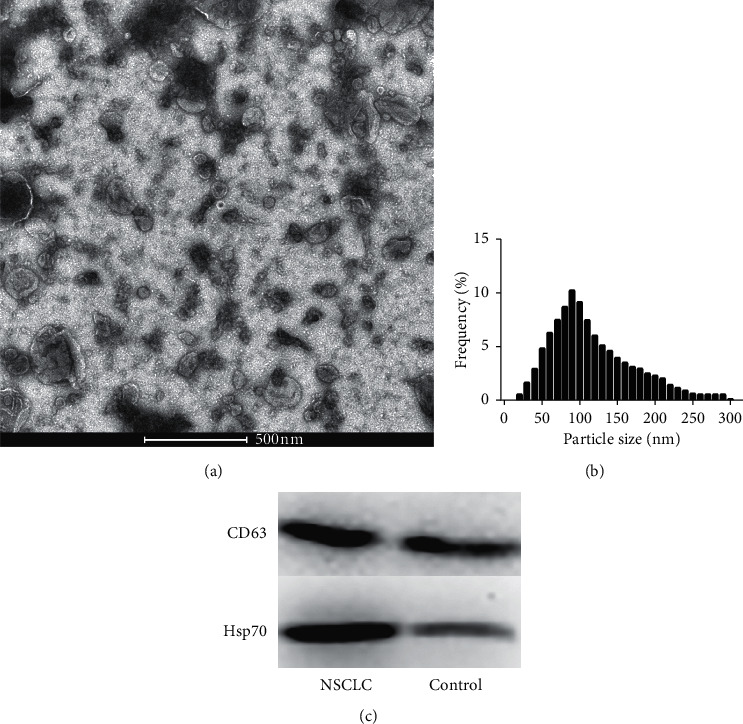
Characterization of plasma exosomes. (a) TEM image showing enriched plasma exosomes in NSCLC patients (scale bar, 100 nm). (b) The size distribution of exosomes was calculated by NanoSight. (c) CD63 and Hsp70 levels in purified plasma exosomes from NSCLC patients were detected by Western blotting.

Plasma exosomes were isolated by lipid nanoprobe (LNP), and TEM indicated that the isolated exosomes had a spherical shape with a diameter of approximately 30–150 nm (Figure 1(a)). NanoSight analysis revealed that the size distribution of exosomes ranged from approximately 50 nm to 200 nm in diameter, with a mode value of 93 nm (Figure 1(b)). Western blot analysis confirmed the expression of the exosomal markers CD63 and Hsp70 (Figure 1(c)).

### 3.2. Expression Levels of Plasma Exosomal miRNAs

**Figure 2:**
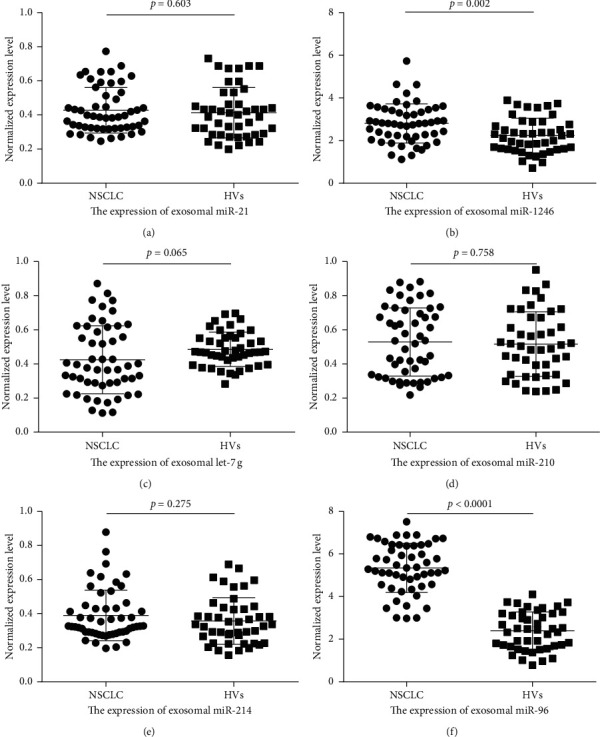
The expression levels of six plasma exosomal microRNAs among NSCLC patients (*n* = 52) and HVs (*n* = 45). Expression of (a) exosomal miR-21, (b) exosomal miR-1246, (c) exosomal let-7g, (d) exosomal miR-210, (e) exosomal miR-214, and (f) exosomal miR-96 in patients with NSCLC and HVs.

The distribution of 6 plasma exosomal miRNAs among the NSCLC patients and HVs is demonstrated in Figure 2. First, we compared the expression levels of the 6 miRNAs in the plasma exosomes from NSCLC patients (*n* = 52) and HVs (*n* = 45) using qRT-PCR. Compared to those in the control group, the levels of plasma exosomal miR-1246 (2.81 ± 0.92) and miR-96 (5.35 ± 1.15) were significantly increased in the NSCLC patients (2.24 ± 0.81 and 2.39 ± 0.89) (*p*=0.002 and *p* < 0.0001). The analysis of expression levels suggested that exosomal let-7g was markedly downregulated in the NSCLC patients (0.42 ± 0.20) compared with the HVs (0.49 ± 0.10), but the difference was not insignificant (*p*=0.065). There were no differences in the levels of plasma exosomal miR-21 (0.43 ± 0.13), miR-210 (0.53 ± 0.20), and miR-214 (0.39 ± 0.15) between the NSCLC and control patients (0.41 ± 0.15, 0.52 ± 0.19 and 0.36 ± 0.14) (*p*=0.063, *p*=0.758 and *p*=0.275, respectively).

### 3.3. Diagnostic Performance of Plasma Exosomal miR-96 and miR-1246

**Figure 3:**
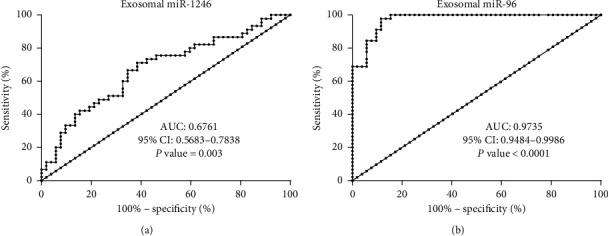
Validation of plasma exosomal miR-96 and exosomal miR-1246 as diagnostic biomarkers for NSCLC. (a) ROC curve of exosomal miR-1246 in NSCLC patients and HVs. (b) ROC curve of exosomal miR-96 in NSCLC patients and HVs.

To determine the diagnostic performance of the two exosomal miRNAs between NSCLC patients and HVs, ROC curve analysis was performed. As shown in Figure 3, the performance of miR-96 yielded area under the ROC curve (AUC) values of 0.9735 (*p* < 0.0001) and miR-1246 AUC = 0.6761 (*p*=0.003). Our results suggest that plasma exosomal miR-1246 and miR-96 may be useful and stable diagnostic biomarkers of NSCLC.

### 3.4. Relationship between Exosomal miRNAs and Resistant NSCLC

**Figure 4:**
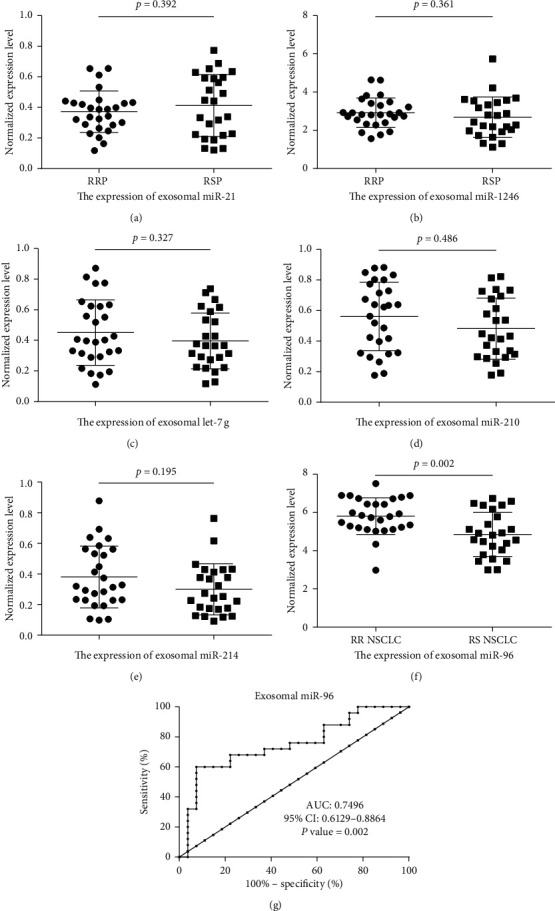
Validation of six plasma exosomal miRNAs as biomarkers for radioresistant NSCLC patients (*n* = 27) and radiosensitive NSCLC patients (*n* = 25). Expression of (a) exosomal miR-21, (b) exosomal miR-1246, (c) exosomal let-7g, (d) exosomal miR-210, (e) exosomal miR-214, and (f) exosomal miR-96 of radioresistant NSCLC patients and radiosensitive NSCLC patients. (g) The ROC curve of exosomal miR-96 in radioresistant NSCLC patients and radiosensitive NSCLC patients. RRP: radioresistant patient, RSP: radiosensitive patient.

Next, we examined the diagnostic ability of the six exosomal miRNAs to distinguish radioresistance in NSCLC patients. As illustrated in Figure 4, only the exosomal miR-96 level of radioresistant NSCLC patients was significantly higher than that of radiosensitive NSCLC patients (*p*=0.002). To determine the diagnostic performance of exosomal miR-96 regarding radioresistance, ROC curve analysis was performed. The performance of exosomal miR-96 yielded an area under the ROC curve (AUC) value of 0.7496 (*p*=0.002). Our results suggest that exosomal miR-96 has the potential to distinguish radioresistant NSCLC patients from radiosensitive NSCLC patients.

### 3.5. Relationship between Exosomal miR-96 and miR-1246 and Clinical Characteristics

**Figure 5:**
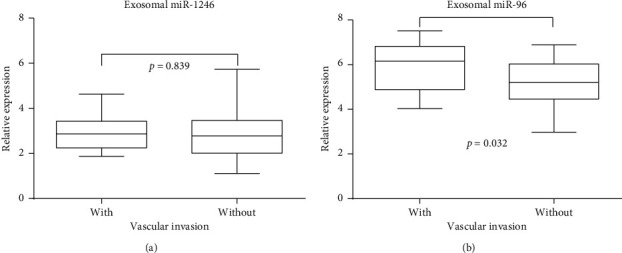
Plasma exosomal miR-1246 and miR-96 expression signature in NSCLC vascular invasion. (a) Relative expression levels of exosomal miR-1246 in NSCLC patients with or without vascular invasion. (b) Relative expression levels of exosomal miR-96 in NSCLC patients with or without vascular invasion.

Since exosomal miR-1246 and exosomal miR-96 could distinguish NSCLC patients from HVs, we further examined their relationship with the clinical, pathological characteristics of NSCLC patients. Correlation analysis showed that exosomal miR-1246 and exosomal miR-96 were not correlated with the history of COPD, chemotherapy, differentiation status, or pathological stage (Table 2). However, as shown in Figure 5, exosomal miR-96 was significantly upregulated in the vascular invasion patients compared to non-vascular filtration patients (*p*=0.035). Exosomal miR-1246 did not show any differences between vascular filtration patients and non-vascular invasion patients (*p*=0.839).

### 3.6. Exosomal miR-96 Is a Candidate Prognostic Factor for the Survival of NSCLC Patients

**Figure 6:**
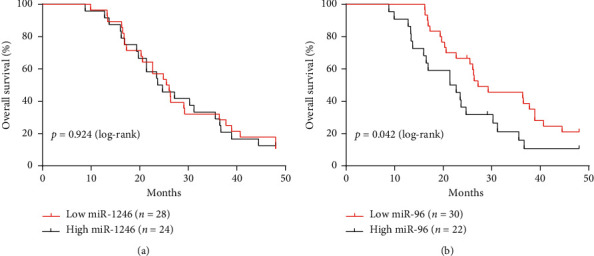
Survival analysis of plasma exosomal miRNAs in NSCLC. (a) The expression of miR-1246 does not show an association trend with OS. (b) Kaplan–Meier curves show that relative expression levels of exosomal miR-96 were significantly associated with OS. *p*-values were calculated using log-rank tests.

A total of 52 NSCLC patients were categorized into high and low exosomal miRNA expression groups using the median miRNA value as the cut-off point. Survival curves of the exosomal miR-1246 and miR-96 estimated by the Kaplan–Meier method are shown in Figure 6. The overall survival of the high exosomal miR-96 expression group was significantly shorter than that of the low exosomal miR-96 expression group, but exosomal miR-1246 did not affect the overall survival of NSCLC patients.

## 4. Discussion

Radioresistance occurs in a high proportion of patients with NSCLC, resulting in poor response to radiation and unfavourable prognosis [21]. Early identification of radioresistance would accordingly guide adjustment of treatment regimens. Cell-derived exosomes contain proteins and nucleic acids and can contribute to intercellular communication [8, 15]. Emerging evidence demonstrates that exosomes are intimately involved in therapeutic resistance, and thus exosomes have the potential to be used to predict radioresistance [11, 22, 23]. Recently, many studies revealed the extraordinary value of exosomal miRNAs as powerful tools to diagnose or even evaluate the prognosis of various cancer diseases [17, 24, 25]. Plasma or serum exosomal microRNAs have been studied as valuable biomarkers of cancer for the following reasons. First, exosomes reflect their tissue origins because they can be immune-isolated using an antibody of a tissue-specific protein on the membrane surface. Second, naked RNAs are easily degraded, whereas exosome-encapsulated RNAs are protected from degrading enzymes in the blood. In our study, we collected exosomal RNA from plasma rather than serum, because it takes a longer time to collect serum and the platelets aggregation, significant serum protein, and exosomes can be encapsulated into the blood clot, resulting in the loss of exosomes.

Unlike conventional genomic approaches that require biopsy methods, exosomal RNAs can be obtained from peripheral blood and other body fluids, which is one of the non-invasive biopsy methods to obtain exosomal RNAs [26–28]. Recently, some reports have shown that exosomal miRNAs are associated with prognosis or are useful diagnostic biomarkers of chemotherapy resistance. Plasma exosomal miR-196a is correlated with poor overall survival and drug sensitivity in the clinic, and miR-196a may serve as a promising predictor of cisplatin resistance in head and neck cancer [29]. Circulating exosomal miR-1290 and miR-375 were shown to be independent predictive markers of overall survival in patients with castration-resistant prostate cancer and improved the predictive value of the standard clinical staging system [17]. However, there are few clinical studies on exosomal miRNAs that predict radioresistance in NSCLC patients. Here, we first reported the existence of exosomal miRNAs in circulating blood of radioresistant NSCLC patients. In this study, we selected miR-21, miR-1246, let-7g, miR-210, miR-214, and miR-96 as diagnostic candidates for validation. We demonstrated that the levels of miR-1246 and miR-96 in plasma exosomes were elevated in NSCLC patients compared to healthy volunteers. The sensitivity and specificity of the exosomal miR-1246 and miR-96 determined by ROC analysis support their use as diagnostic biomarkers. Their expression levels had a significant improvement in NSCLC survival prediction compared to traditional clinical prediction methods.

Next, we examined the potential diagnostic significance of six exosomal miRNAs to differentiate radioresistant patients from radiosensitive patients with NSCLC. Only the exosomal miR-96 level of radioresistant NSCLC patients was significantly higher than that of radiosensitive patients. The performance of miR-96 yielded an area under the ROC curve (AUC) value of 0.7496 (*p*=0.002). Our results suggest that the expression of miR-96 demonstrated high sensitivity and specificity and clearly distinguished radioresistant patients from radiosensitive patients. Another interesting finding in this study was that increased exosomal miR-96 was associated with vascular invasion in NSCLC patients, while miR-1246 did not show any differences. Indeed, exosomal miR-96 showed high sensitivity for vascular filtration. However, the levels of exosomal miR-96 and miR-1246 were not correlated with the history of COPD, chemotherapy, differentiation status, or pathological stage. These findings suggest that exosomal miR-96 is a suitable biomarker for the detection of vascular filtration in NSCLC.

We determined that high expression of exosomal miR-96 was associated with poor prognosis using a Kaplan–Meier survival curve. MiR-96 is one of the most frequently studied miRNAs in cancer [24, 29–31]. Recently, accumulating evidence has shown that there is a significant increase in the expression of miR-96 in chemo- or radioresistant tumours [24, 29]. Indeed, this study confirmed that miR-96, which is often deregulated in cancer, acts as an oncogene miRNA in NSCLC. It was shown that high levels of miR-96 induced cell proliferation and cell cycle progression by targeting FOXO1, which is a central component of the AKT/FOXO1/Bim pathway. Furthermore, exosomal miR-96 promoted lung cancer progression by targeting LMO7. The miR-96-LMO7 axis may be a therapeutic target for lung cancer patients. Mark et al. demonstrated that miR-96 promoted prostate cancer progression through the RAR*γ* signalling axis, which regulates androgen. These significant characteristics of the miR-96 family and the close connection between exosomes and miRNAs contained in exosomes guided the research design of this study.

## 5. Conclusion

In this study, we selected miR-21, miR-214, miR-1246, miR-210, miR-96, and let-7g as diagnostic candidates for validation. Among these candidates, plasma exosomal miR-96 and miR-1246, which were upregulated in patients with NSCLC compared to healthy volunteers, were independent diagnostic biomarkers of NSCLC. We also found that increased exosomal miR-96 was related to vascular invasion in NSCLC patients. More importantly, miR-96 was a potential diagnostic biomarker of radioresistant NSCLC and a prognostic biomarker of NSCLC. The results of this study need to be confirmed in a large cohort of NSCLC patients. In addition, further studies are needed to validate the possible application of exosomal miR-96 for survival prediction and to explore its potential mechanisms in lung cancer radioresistance and progression.

## Figures and Tables

**Table 1:** Relationships between miR-1246 and miR-96 expression (qRT-PCR) in NSCLC plasma exosomes and various clinicopathological variables.

Variable	Total	miR-96 expression	*p* value	miR-1246 expression	*p* value
Surgery treatment					
Yes	26	5.577 ± 1.215	0.223	2.703 ± 0.732	0.416
No	26	5.113 ± 1.061		2.909 ± 1.081	

Differentiation status					
High/moderate	27	5.215 ± 0.863	0.441	2.727 ± 0.729	0.554
Poor	25	5.465 ± 1.375		2.880 ± 1.076	

Chemotherapy					
Yes	47	5.397 ± 1.099	0.533	2.792 ± 0.896	0.839
No	5	5.150 ± 1.38		2.857 ± 1.049	

History of COPD					
Yes	41	5.410 ± 1.120	0.217	2.880 ± 0.895	0.074
No	11	4.735 ± 1.428		2.107 ± 0.953	

Vascular invasion					
Yes	14	5.896 ± 1.121	0.035	2.906 ± 0.832	0.638
No	38	5.142 ± 1.111		2.769 ± 0.958	

**Table 2:** Clinical characteristics of patients and healthy individuals of two sets.

Variable	NSCLC (52)		HV (45)	*p* value
	RRP (27)	RSP (25)		
Age (years)	53.3 ± 12.1	56.8 ± 15.5	55.7 ± 13.6	0.999
Sex (male/female)	18/9	15/10	26/19	0.774
Smoking	22	19	34	0.862
Pathological stage				
II	3	2	—	0.777
III	15	13		
IV	8	10		

Surgery treatment	11	13	NA	0.578

Differentiation status				
High	3	2	NA	0.923
Median	12	10		
Low	12	13		

Chemotherapy	25	22	NA	0.662
History of COPD	20	21	NA	0.503
Vascular invasion	9	5	NA	0.355

RRP: radioresistant patient; RSP: radiosensitive patient; HV: healthy volunteer.

## Data Availability

The data used to support the findings of this study are available from the corresponding author upon request.
